# Effects of Isometric Scaling on Vertical Jumping Performance

**DOI:** 10.1371/journal.pone.0071209

**Published:** 2013-08-01

**Authors:** Maarten F. Bobbert

**Affiliations:** MOVE Research Institute Amsterdam, Faculty of Human Movement Sciences, VU University Amsterdam, Amsterdam, The Netherlands; The University of Queensland, Australia

## Abstract

Jump height, defined as vertical displacement in the airborne phase, depends on vertical takeoff velocity. For centuries, researchers have speculated on how jump height is affected by body size and many have adhered to what has come to be known as Borelli’s law, which states that jump height does not depend on body size per se. The underlying assumption is that the amount of work produced per kg body mass during the push-off is independent of size. However, if a big body is isometrically downscaled to a small body, the latter requires higher joint angular velocities to achieve a given takeoff velocity and work production will be more impaired by the force-velocity relationship of muscle. In the present study, the effects of pure isometric scaling on vertical jumping performance were investigated using a biologically realistic model of the human musculoskeletal system. The input of the model, muscle stimulation over time, was optimized using jump height as criterion. It was found that when the human model was miniaturized to the size of a mouse lemur, with a mass of about one-thousandth that of a human, jump height dropped from 40 cm to only 6 cm, mainly because of the force-velocity relationship. In reality, mouse lemurs achieve jump heights of about 33 cm. By implication, the unfavourable effects of the small body size of mouse lemurs on jumping performance must be counteracted by favourable effects of morphological and physiological adaptations. The same holds true for other small jumping animals. The simulations for the first time expose and explain the sheer magnitude of the isolated effects of isometric downscaling on jumping performance, to be counteracted by morphological and physiological adaptations.

## Introduction

Jumping is important for survival of many animals because it helps them to catch preys or escape from predators [1]. Jump height (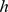), defined as vertical displacement of the centre of mass (CM) in the airborne phase, has been found to vary substantially among differently sized primate species. For example, *h* is about 0.33 m in a 100 g grey mouse lemur (*Microcebus murinus*) [2], up to about 2 m in a 300 g bushbaby (*Galago senegalensis*) [3], up to 0.7 m in a 34 kg bonobo (*Pan paniscus*) [4] and typically about 0.4 m in a 75 kg human [5]. From the perspective of functional morphology, it is interesting to compare jumping performance among species. *Galago senegalensis* seems to be *hors catégorie*, because it outperforms other mammals in both absolute and relative terms, but how does *Microcebus murinus* perform compared to humans? For centuries, the consensus in the literature has been that comparisons should be made in terms of absolute jump height, so *Microcebus* is not a good jumper compared to humans. However, in relation to body size *Microcebus* does a much more impressive job than humans. Is absolute jump height a fair measure to compare jumping performance of differently sized primate species?

In the literature, various propositions can be found on how body size affects jump height. The first proposition is that body size does not affect jump height at all. Perhaps most current scientists will not adhere to this proposition, but it has played a dominant role in history and hence is a good starting point here. The proposition has come to be known as Borelli’s law, because Borelli, in his book *De Motu Animalium* published in 1680 [6], was the first to suggest that takeoff speed should be the same regardless of animal size [7]. A few years later, in 1687, Newton formulated the laws of classical mechanics in *Philosophiae Naturalis Principia Mathematica* [8], and many authors have used these laws to reason why isometrically scaled animals should have the same jump height (e.g., [9]–[11], for specific formulations see [7]). The reasoning is as follows. To jump to a given *h*, an animal must achieve a certain vertical takeoff velocity of CM (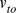), which corresponds to kinetic energy (*E_kin,to_*) equal to 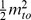, where *m* is body mass. Neglecting air resistance, *E_kin,to_* is transformed during the airborne phase into potential energy 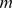, where *g* is the acceleration due to gravity, so:
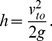
(1)


For the remainder of this paper, it is helpful to write this as:
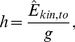
(2)where 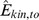 is *E_kin,to_* expressed per kg body mass. If we assume that there is no difference among animals in the amount of work produced per kg of muscle, and no difference in the amount of muscle mass relative to body mass, then each animal should produce the same amount of work per kg body mass (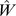), leading to the same 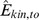 and hence to the same *h* (equation 2). According to this proposition, it is fair to compare jumping performance of differently sized primate species in terms of absolute jump height.

Recently, Scholz et al. [7] showed that if differently sized animals produced the same 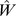, small animals should actually be expected to jump higher than big animals. During the push-off, CM gains potential energy equal to 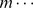, where *s* is the vertical displacement of CM from initial height to takeoff height. If big animal B is isometrically downscaled to small animal S by a factor *L*, *s* will scale by *L* too. Hence, if animal S and animal B produce the same 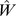, animal S will need a smaller fraction of 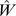 to raise CM to the takeoff height, has a greater fraction of 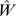 available for 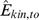, and hence achieves greater *h* (equation 2). Accordingly, under the assumption that all animals produce the same 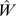, comparing jumping performance in terms of *h* is unfair to big animals. This proposal will be referred to as the revised version of Borelli’s law. Note that the definition of jump height is crucial here. If jump height were defined as the vertical displacement of CM relative to the lowest height of CM during the jump, which is proportional to the total change in effective energy per kg body mass (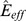, the sum of potential energy and kinetic energy due to the vertical velocity of CM), then Borelli’s law would still hold. In that case, however, simply standing up from a crouched position would qualify as a jump, which seems unacceptable.

Although the argumentation presented above is mechanically straightforward, there is reason to question that small animals should be able to jump higher than big animals. Motion of CM is the result of rotations of body segments. If the motion pattern were invariant (IMP), i.e. if differently sized animals produced the same segment rotational kinematics over time, *v_to_* would simply be proportional to *L*, and *h* to 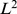 (equation 1). It is known that when small animals jump, they produce higher accelerations than large animals [12], and they could in principle achieve higher 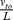 and 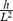 than large animals. However, these higher accelerations in themselves require explanation, and they may not be sufficient to cause small animals to jump higher than big animals in absolute terms.

The revised version of Borelli’s law holds under the assumption that all animals produce the same 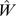 during the push-off. Bennet-Clark [12] reasoned that under a different assumption, namely that all animals produce the same peak power per kg muscle mass, animal S will achieve smaller *h* than animal B. Bennet-Clark’s argument was as follows. Producing the same 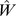 during the push-off requires animal S to produce a higher peak power per kg body mass during the jump than animal B. After all, animal S has shorter limbs and smaller *s* for acceleration of CM (*a*) than animal B. Assuming that *a* is constant during the push-off, 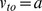, where *T* is push-off duration, and 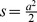, from which it that can be derived that 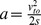. If *a* is constant, peak power occurs at takeoff and equals 
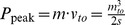
 (note that 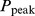 as defined by Bennet-Clark is actually the peak rate of change of kinetic energy due to vertical velocity of CM). Combining this with equation 1 yields
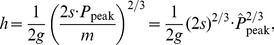
(3)where 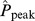 is 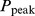 per kg body mass. According to Bennet-Clark [12] it is reasonable to assume that 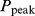 is proportional to muscle mass, which in isometric scaling is proportional to *m*. Since *s* is proportional to *L*, *h* is proportional to 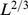 under this assumption. Bennet-Clark’s reasoning is another way of saying that if 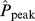 is the limiting factor, 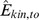 is proportional to 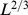, so that animal S should be expected to jump less high than animal B. Therefore, a comparison in terms of *h* is unfair to animal S.

Comparing jumping performance in terms of *h* may also be unfair to small animals for reasons related to muscle physiology, and this is the topic of the present paper. Firstly, to achieve a given 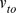, animal S needs higher segment angular velocities than animal B. After all, the velocity of CM is determined by the angular velocities of body segments, and if the segments are shorter the angular velocities need to be higher for the same absolute velocity of CM. This will require animal S to traverse the range of joint motion at higher angular velocities and, because muscle moment arms and muscle fibre lengths scale by *L*, contractile elements (CE) will shorten at higher relative velocities (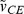, i.e. CE velocity expressed in optimum CE-lengths per second). At higher 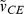, CE will produce less relative force (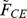, i.e. CE force as fraction of maximum isometric force) and less 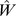 because muscle force drops monotonically with shortening velocity according to the force-velocity relationship [13]. Secondly, it takes time to develop active state. Active state, which has been defined as the amount of Ca^++^ bound to troponin [14], affects the number of cross-bridges attached and hence 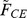. If animal S traverses the range of motion at higher angular velocities and hence in less time than animal B, a relatively larger part of the range of CE-shortening will be travelled at submaximal active state and submaximal 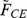 in animal S, and this will also detract from 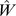 produced during the push-off [15], [16].

The propositions on how body size affects jump height presented above are all based on simplifying assumptions, for example that 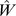 over the push-off is independent of size [10], or that 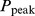 during the jump is proportional to body mass and hence independent of size [12]. Moreover, although it will be clear from the reasoning presented above that both the force-velocity relationship and the rise time of active state present a disadvantage for small animals, the magnitude of the effects is difficult to predict. It would be helpful, therefore, to study the effects of isometric scaling using a biologically realistic musculoskeletal model. Alexander [17] studied jumps of humans, bushbabies and locusts with a realistic musculoskeletal model that included series elastic structures and muscle forces depending on length and velocity. However, he made three separate models, each with species-specific morphological and physiological parameters, and from his simulation results it is impossible to tease apart the possible effects of pure isometric scaling from the effects of inter-species differences in morphology and physiology.

The purpose of the present study was to quantify the effects of pure isometric scaling on 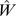, *h* and other mechanical and physiological variables relevant for jumping, using a biologically realistic model of the human musculoskeletal system.

## Methods

### Musculoskeletal Model

**Figure 1:**
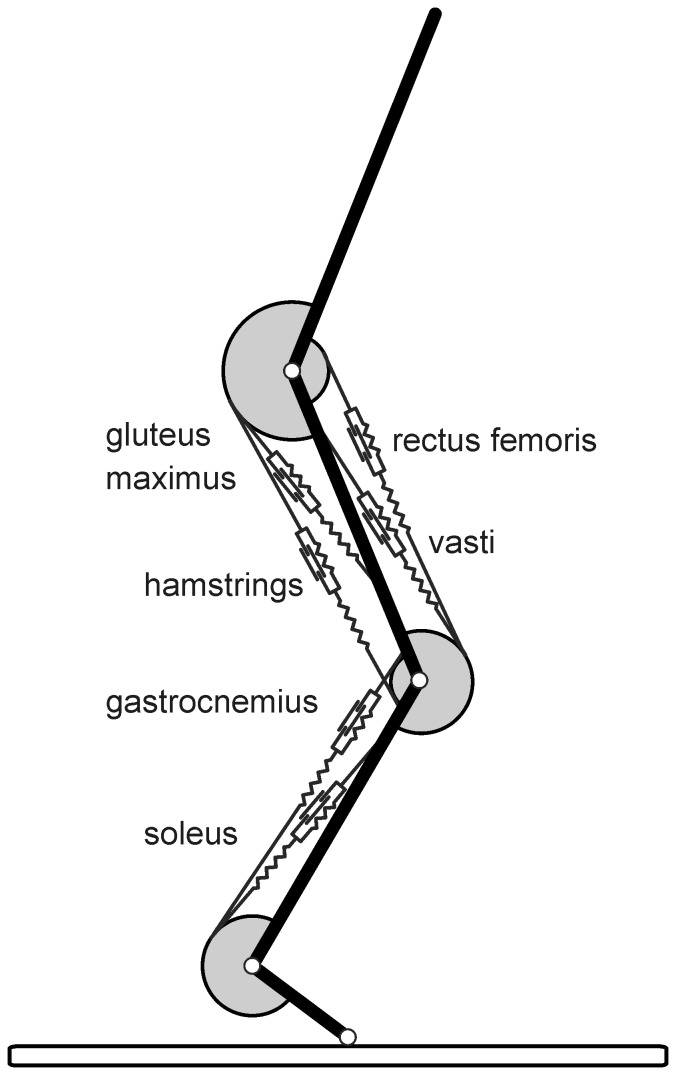
Musculoskeletal model used for simulations. Note that because of space limitations in the figure, the moment arms of the muscle-tendon complexes at the joints (gray spacers at the joints) have not been drawn to scale; the actual moment arm values are presented elsewhere [18].

Vertical squat jumps were simulated using a musculoskeletal model capable of successfully reproducing human vertical jumps [18], [19] (Fig. 1). It comprised four body segments, actuated by six major muscle tendon complexes (MTCs) of the human lower extremity. Each MTC was represented by a Hill type unit, comprising contractile element CE, series elastic element SEE and parallel elastic element PEE. Forces of SEE and PEE quadratically increased with SEE elongation only, while force of CE (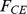) depended on length of CE (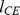), velocity of CE (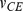) and active state [20]. Active state, in turn, dynamically depended on muscle stimulation over time (*STIM(t)*).

### Simulation of Jumps

**Figure 2:**
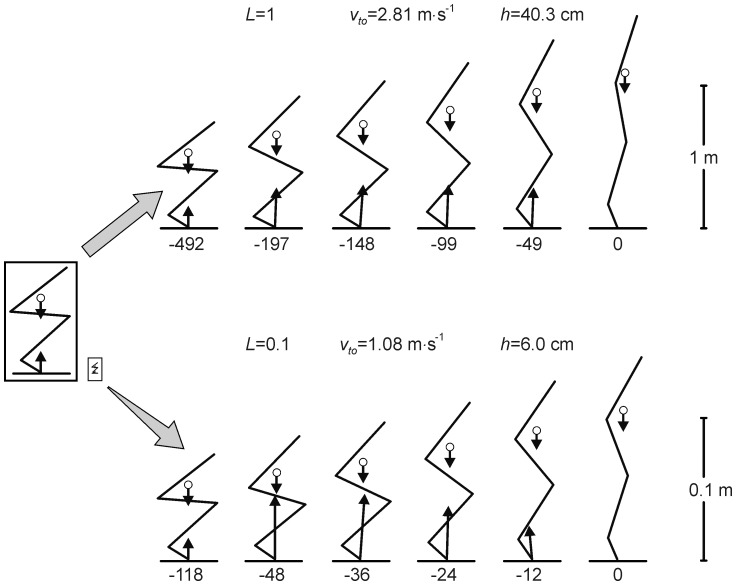
Stick diagrams for maximum height jumps of models with isometric scale factors of 1 (*L* = 1) and 0.1 (*L* = 0.1). Arrows pointing upward represent the ground reaction force vector and originate in the centre of pressure; arrows pointing downward represent the force of gravity and originate in the centre of mass (CM, open circles). Numbers below sticks indicate time in ms relative to takeoff. The leftmost stick diagrams represent the initial equilibrium posture, the other stick diagrams are spaced by one-tenth of the duration of the push-off. 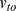: vertical takeoff velocity of CM, *h*: jump height.

Squat jumps were simulated from the initial posture shown in Fig. 2, which is considerably crouched for humans [19]. Initial *STIM* levels were set such that the model was in equilibrium. During the jump, *STIM* of each MTC was only allowed to increase linearly towards its maximum at a reference rate of 5/s [19], and this increase started at a *STIM* onset time. The combination of *STIM* onset times that maximised the height achieved by CM was found using a genetic algorithm [21] for each value of *L*. In order to quantify the effect of active state dynamics, we also optimized *STIM* onset times for a model in which *STIM* increased instantaneously to its maximum at *STIM-*onset time and the rate constant of excitation dynamics had been boosted by a factor of 100.

It might occur to the reader that humans and most animals tend to make countermovement jumps and not squat jumps. Making a countermovement has the advantage that active state and force can be built up during the downward motion of CM rather than during the push-off [15]. However, this advantage becomes negligible when active state increases rapidly [15], and it can safely be said that the outcome of the present study would have been the same if countermovement jumps had been simulated.

### Scaling of the Model

The author was interested in animals ranging in size from humans to *Microcebus*, with mass being used as the variable for scaling. The human musculoskeletal model, with a mass of 82 kg, served as reference model (*L* = 1). *Microcebus* has a mass of only 90–100 g [2], which is about one-thousandth of the reference mass. Therefore, *L* was chosen to run from 1 to 0.1 (i.e. 0.001^1/3^), in 30 steps. All body segment lengths, distances of segmental mass centres to segment ends, and muscle moment arms, were scaled by *L*, all masses by 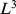, and all moments of inertia for rotation about the segmental mass centre, with 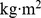 as unit, by 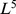. Lengths of CE, SEE and PEE were scaled by *L* and their forces, which depend on physiological cross-sectional areas, by 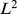. Note that for all scales, the maximum shortening velocity was 12.7 optimum CE lengths per second. Because muscle fibre length scaled by *L*, the maximum shortening velocity of muscle fibres in absolute terms, i.e. in m/s, also scaled by *L*. The specific tension of the muscles in the model was taken to be 0.25 MPa and the theoretical maximal power output 367 W per kg of muscle tissue [22], which gave the model a theoretical maximal CE power output of 60 W per kg of body mass independent of scale.

## Results

**Figure 3:**
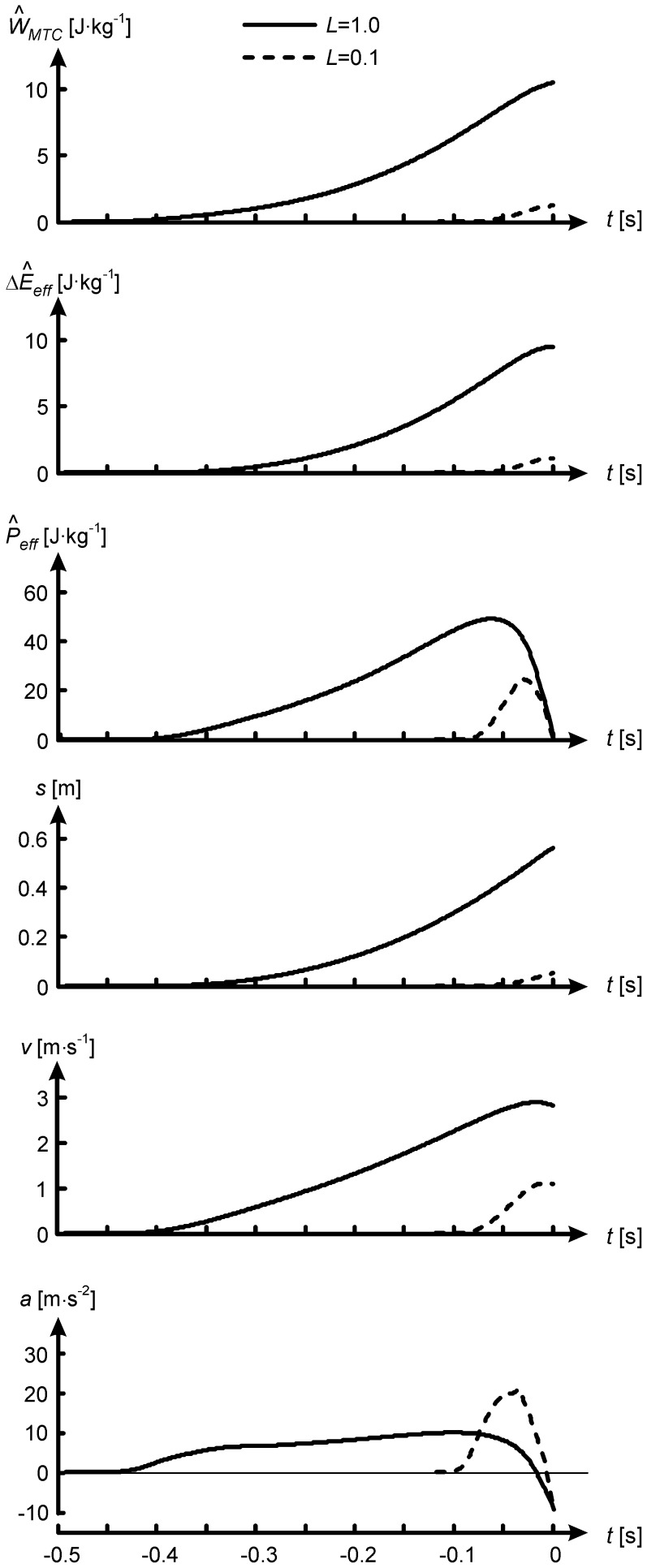
Kinematic, energetic and work variables as function of time for models with isometric scale factors of 1 (*L* = 1) and 0.1 (*L* = 0.1). 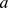
 is vertical acceleration of centre of mass (CM), 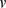 vertical velocity of CM, and 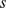 vertical displacement of CM. 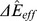 is increase in effective energy during push-off relative to the start of the jump, 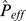 rate of change of 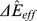, and 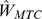 work of muscle-tendon complexes, all expressed per kg body mass as indicated by caret over variables. Time (*t*) is expressed relative to takeoff (*t* = 0).

Figure 2 presents for *L* = 1 and *L* = 0.l models stick diagrams including ground reaction force vectors, and values for 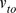 and *h*, and Fig. 3 shows time histories of relevant variables for *L* = 1 and *L* = 0.1. The first observation is that the duration of the push-off in the *L* = 0.l model is only about 25% of that in the *L* = 1 model. The second observation is that the acceleration of CM increases with miniaturization, but that takeoff velocity and hence jump height nevertheless decrease. The third observation is that, although the theoretical maximum power output of the muscles per kg body mass was independent of scale, the mean and peak values of 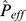 (the rate of change of 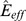) drop at small values of *L*.

**Figure 4:**
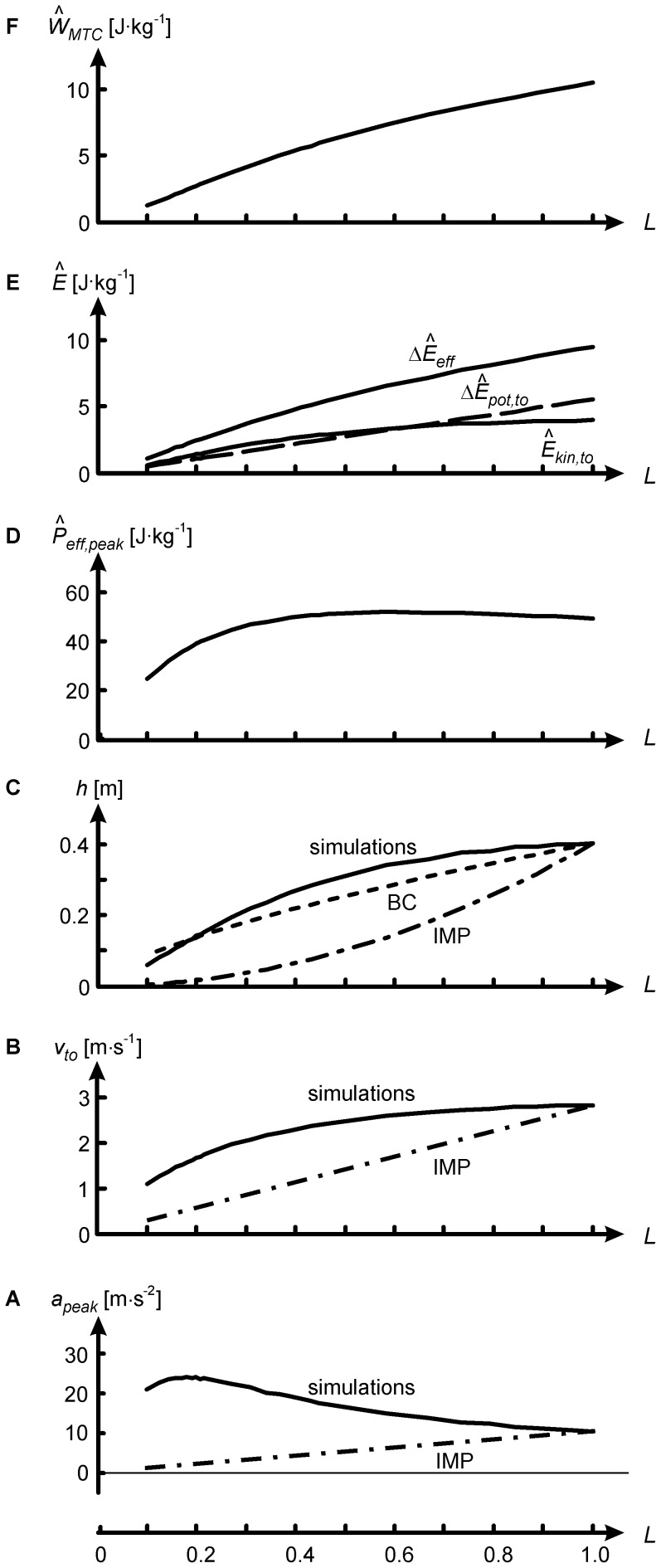
Kinematic, energetic and work variables as function of isometric scale factor *L*. 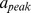
 is peak vertical acceleration of centre of mass (CM), 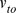 vertical velocity of CM at takeoff, and *h* (jump height) is vertical displacement of centre of mass (CM) in the airborne phase. 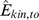 is kinetic energy due to 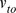, 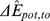 increase in potential energy during push-off, 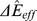 increase in effective energy during push-off (sum of 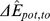 and 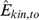), 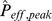 peak rate of change of 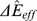 during the push-off, and 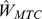 work of muscle-tendon complexes, all expressed per kg body mass as indicated by caret over variables. IMP: Invariant Motion Pattern, i.e. values as they would be if segment angles over time were the same as in reference model (*L* = 1). BC: dependence of *h* on *L* predicted by Bennet-Clark [12] (equation 3).

Fig. 4 (A–C) shows how kinematic variables changed over the investigated range of *L*. Dash-dotted lines in Fig. 4A–C represent outcomes as they would be if the Motion Pattern were Invariant (IMP), i.e. if a model with L<1 had the same segment angles, angular velocities and angular accelerations over time as the *L* = 1 model. Under IMP, *a* and *v_to_* would be proportional to *L*, and *h* to 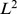 (equation 1). In isometrically downscaled models, however, peak *a*, 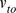 and *h* exceeded the values corresponding to IMP. Thus, in relation to body size, i.e. in terms of 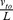 and 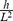, isometrically downscaled models performed better than the reference model. However, with downscaling the duration of the push-off phase became less, and in absolute terms lower 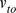 and *h* were reached. The model jumped 40 cm when human-sized, only 10 cm when miniaturized to the size of a 300 g bushbaby, and only 6 cm when miniaturized to the size of *Microcebus*. Thus, with isometric scaling, small animals jump less high than big animals, in contrast to Borelli’s law and its revised version.

Scaling *h* by 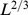 as proposed by Bennet-Clark ([12], dashed curve in Fig. 4C) overestimated *h* of the *L* = 0.1 model by only 2.7 cm, but overall the relationship between *h* and *L* could not be fitted well with 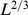, nor with any other power of *L*. Bennet-Clark’s estimation builds on a constant peak power per kg body mass (equation 3), but the actual peak 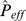 reached during the jump decreased from 49 W/kg at *L* = 1 to about 25 W/kg at *L* = 0.1, even though the theoretical maximum power output of the muscles per kg body mass was kept constant across scales. The reader might point out that 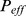 is not equal to the power output of contractile elements (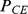) summed over all MTCs. On the one hand, 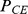 of an MTC may differ from the power output of the MTC as a whole (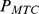) because of the presence of series elastic elements; for example, when muscle force drops during the final part of the push-off these elements recoil, causing MTC shortening velocities to be higher than muscle fibre shortening velocities, and hence causing 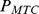 to be higher than 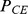 [23], [24]; this is known as ‘catapult action’. On the other hand the total 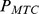 of the model will differ from 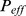 because of power flow to non-effective terms, such as segment rotational power [25]. Despite these caveats, however, the peak of 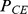 summed over all MTCs, expressed per kg body mass, showed the same behaviour as peak 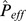, dropping from 48 W/kg at *L* = 1 to 24 W/kg at *L* = 0.1 (results not shown). As mentioned above, the ‘catapult action’ causes peak 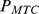 to be higher than peak 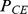 [23], [24]. This action is very important for performance in jumping [25], but it disqualifies peak power output as measure for the performance of muscle tissue. In the literature, comparisons among different animals are therefore also made in terms of mean power output over the push-off phase. During the push-off phase, the model produced a mean 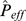 of 19 W/kg when human-sized, 13 W/kg when miniaturized to the size of a 300 g bushbaby, and 9 W/kg when miniaturized to the size of *Microcebus*. For mean 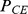 per kg body mass, summed over all MTCs, these values were 21, 14 and 10 W/kg, respectively.

**Figure 5:**
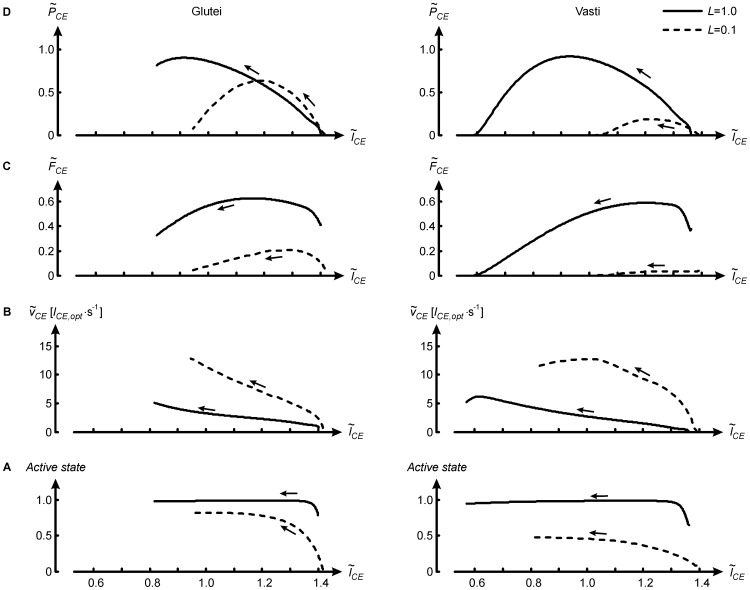
Explanation for reduced work output per kg of glutei (left panels) and vasti (right panels) with isometric downscaling. Force, velocity and active state of contractile elements (CE) of glutei and vasti have been plotted as function of normalised CE-length (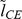) for models with isometric scale factor *L* equal to 1 (reference model) and 0.1. 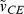 is CE shortening velocity expressed in optimum CE-lengths (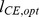) per second, 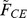 is CE force as fraction of maximum isometric force, and 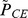 is CE power output as fraction of its maximum according to the force-velocity relationship. Arrows indicate the direction of time. When *L = *0.1, CE work per kg (proportional to surface under curves in A and D) is less because 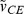 is higher and active state is lower than when *L* = 1.

According to equation 2 the drop in *h* with reduction of *L* (Fig. 4C) corresponds to a drop in 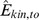. Figure 4F shows that the latter was due to a drop in work per kg body mass of muscle-tendon complexes (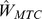). In the simulated squat jumps, 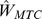 produced during the push-off phase depended for more than 99% on contractile element work per kg body mass (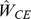), where 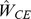 of a given MTC is proportional to the integral of 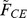 to normalised CE-length (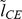). 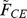 at given 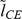 depends on 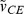 and active state. To analyze differences in 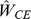 of a given MTC it is therefore helpful to plot 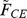, 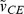 and active state as function of 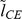 [25]. This has been done in Fig. 5 for glutei and vasti, which at *L* = 1 contributed 35% and 30% to total 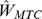, respectively. At *L* = 0.1, 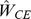 of glutei was reduced compared to *L* = 1 for two main reasons, as can be explained with the help of the left panels of Fig. 5A–C (note that 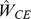 is proportional to the surface under the curve in Fig. 5C). First, in *L* = 0.1, 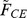 was reduced because of the force-velocity relationship: 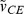 was higher at each 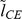 (Fig. 5B). Second, in *L* = 0.1, 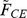 was reduced because active state was lower at each 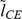 (Fig. 5A); in the model the increase in active state was fixed over time, but the range of 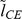 was traversed in less time. Note that the lower active state was also part of the reason why power output of CE reached a smaller peak value at *L* = 0.1 (Fig. 5D). It can also be seen in Fig. 5 that at *L* = 0.1, CE shortened over a smaller range, because at takeoff joints were less extended than in the reference model (Fig. 2). However, takeoff occurs *because* the muscle forces become insufficient [26], so the reduced range of motion in *L* = 0.1 is a *consequence* of the higher 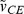 and lower active state in *L* = 0.1. The explanation for the reduced 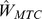 of vasti at *L* = 0.1 is essentially the same as that for the reduced 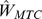 of glutei, but the effect of the force-velocity relationship was even more devastating (Fig. 5, right panels); 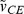 increased almost immediately after the start of shortening to values at which only small 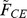 was produced.

What is the relative importance of these two complications of isometric scaling? When muscle active state was allowed to increase almost instantaneously to its maximum and *STIM(t)* was re-optimised, *h* increased by only 0.2 cm at *L* = 1 and by only 2.5 cm at *L* = 0.1, suggesting that muscle dynamics constitute a much bigger complication than activation dynamics.

## Discussion

The purpose of the present study was to quantify the effects of pure isometric scaling on 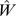, *h* and other mechanical and physiological variables relevant for jumping, using a biologically realistic model of the human musculoskeletal system. In the simulated jumps presented in this paper, angular and linear accelerations increased when a human model was isometrically downscaled. The increased angular accelerations led to higher angular velocities and, in relation to body size, downscaled models performed better than the reference model. However, in absolute terms 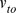 and hence 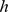 dropped because 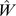 became less, rather than remaining constant as had been assumed by proponents of Borelli’s law (e.g., [9]–[11]). Bennet-Clark [12] had already predicted that 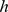 would decrease with size under the assumption that 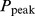 was proportional to body mass. However, the relationship between 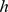 and body size borne out by the present simulations was different from the one that Bennett-Clark had proposed [12]. Although in the model the theoretical maximum power output of the muscles per kg body mass was kept constant across scales, the mean and peak values of 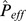 (the rate of change of 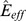) dropped substantially at small values of *L* (Fig. 3, Fig. 4D). Below, I will first explain these findings and then elaborate on their relevance for the study of functional morphology and evolution of jumping animals.

We have seen that with reduction of *L*, segment angular accelerations and linear accelerations increased (Figs. 3–4). The increased angular accelerations led to higher angular velocities and, in relation to body size, i.e. in terms of 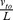 and 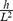, the downscaled model performed better than the reference model; in other words, it performed better than under IMP, (Fig. 4A–C, dash-dotted lines). Why did segment angular accelerations increase with miniaturization? Translation of CM occurs because muscle moments rotate body segments against gravitational moments. Maximal muscle moments at the joints are proportional to 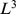 because they are the product of muscle force proportional to 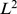 and moment arms proportional to *L*. They act against moments due to gravity proportional to 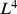 (the product of mass proportional to 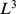 and moment arms relative to joints proportional to *L*) and cause angular accelerations of segments with moments of inertia proportional to 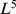. This explains why angular accelerations, and the ensuing angular velocities, increase as size decreases. Clearly, as was already pointed out elsewhere in general terms [27], [28], an isometrically downscaled animal is relatively strong and moves relatively fast. However, as revealed by the simulations in this study, these positive effects are counteracted by negative effects: compared to the muscles of the reference model, the muscles of an isometrically downscaled model have less time to build up active state and, more importantly, are hampered more in their force production by the force-velocity relationship, leading to a decrease in 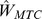, in 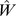, in 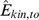 and hence in *h* in absolute terms. In conclusion, a small animal that is an isometrically downscaled version of a big animal achieves lower *h,* and this is largely due to muscle dynamics. The simulations reveal the sheer magnitude of the effect of isometric scaling on *h*: the model jumped 40 cm when human-sized, and only 6 cm when miniaturized to the size of *Microcebus*. This puts the 33 cm jump height of *Microcebus* [2] in a different perspective: *Microcebus* is not performing poorly compared to humans, as Borelli and his followers would have concluded, but instead jumps to more than five times the height expected on the basis of isometrically downscaling a human body. The same is true for other small mammals such as rats, which also seem to be able to achieve jump heights of 50 cm or more [29].

In a general sense, the results of the present study merely reiterate what had already been claimed by Bennet-Clark ([12]). However, Bennet-Clark’s predictions (equation 3, Fig. 4C) were purely based on the argumentation that a smaller animal has a smaller distance over which to accelerate CM and hence a smaller push-off time; they took into account neither the positive effect of isometric downscaling on relative strength explained above, nor the negative effects of the force-velocity relationship on actual peak power output (Fig. 4D, Fig. 5D) and work (Fig. 4E) during the jump. The present study quantified the effects of pure isometric scaling on jumping performance using forward dynamic simulations with a realistic musculoskeletal model. Here, it was not necessary to adopt Bennet-Clark’s assumption that the shapes of time-histories of force, velocity and hence power are consistent across scales, which they are not (Fig. 3). Even for current scientists who did not adhere to Borelli’s law in the first place and considered its role in the present paper as that of a straw man, the sheer magnitude of the effect of pure isometric scaling on *h* and other relevant variables, as borne out by the simulations (Fig. 4), may still come as a surprise.

The simulation model used in this study is realistic in that it takes into account the fundamental properties of the components of the musculoskeletal system and in terms of parameter values represents a human musculoskeletal system. However, after miniaturization it obviously does not represent the musculoskeletal system of small primates. There are many morphological and physiological differences that may help small primates to jump higher than a downscaled human model. Let us address a few of these differences and their functional implications, armed with the insights gained from the simulations. First of all, small jumping primates may have relatively muscular legs; for example, the muscle mass contained in both legs together is about 25% of total body mass in *Galago senegalensis* [3] and only about 17% in humans [30]. It will be obvious that this benefits 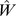, 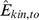 and hence *h*. Second, small primates have relatively long leg segments, including an elongated metatarsal segment ([17]), which benefits the transfer from joint angular velocities to vertical velocity of CM. Third, small primates have relatively short muscle moment arms [28], which benefits the transfer from 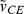 to angular velocities. Having relatively long muscle fibres would tend to reduce 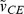 itself, but the author has not come across any comparisons of relative muscle fibre length among differently sized primates in the literature. Fourth, an important role has been claimed for compliant structures in series with muscle fibres in the vasti of *Galago senegalensis* [3]. It is possible that the ‘catapult action’ of these structures contributes more to jumping performance in small jumping primates than in humans. However, this action depends on precisely how moment arms vary with joint angles [31], and therefore its quantification requires simulations with detailed species-specific musculoskeletal models that, unfortunately, are currently not available. Fifth, small animals have equally long myosin filaments as large animals (1.60 µm, [32]) but shorter actin-filaments (e.g., 1.04 µm in rats, 1.16 µm in Rhesus monkeys, 1.27 µm in humans, [32]). Thus, in a small animal, a unit of muscle fibre length will have more sarcomeres in series and, at a given rate of sliding of actin relative to myosin, higher velocity and power output than in humans, all else remaining equal. Sixth, small jumping animals tend to have higher percentages of fast twitch fibres in important leg extensors such as vastus lateralis (more than 95% in *Microcebus*, [33], and 100% in bushbabies, [34]) than humans (less than 60%, [35]), which obviously benefits the power output per kg muscle tissue. Seventh, the maximal shortening velocity of muscle fibres and the rate of force development are higher in small animals than in large animals (e.g., [36]–[39]) because of differences in intrinsic contractile properties and myofibrillar protein composition [36], [39]. The latter variations are referred to in the literature as ‘effects of scaling’ (e.g., [36], [37]), but this is confusing because isometric scaling does not affect these variables directly. Rather, isometric scaling directly affects potential performance, as clearly demonstrated in the current study, and morphological and physiological adaptations may occur that partly or completely counteract the variations caused by isometric scaling, thereby determining actual performance. For example, small animals may jump high despite the performance-limiting effects of being small revealed in this study, by virtue of adaptations causing their muscles to be very fast, and/or large animals may jump high by virtue of the performance-enhancing effects of being large revealed in this study, and therefore can afford adaptations causing their muscles to be slower and metabolically cheaper. We are still a long way from understanding the effects of morphological and physiological differences among different animals on locomotor performance, but the results of the current study indicate that they compensate for major effects of isometric scaling (Fig. 5A). Clearly, the effects of isometric scaling on jumping performance, as revealed here by simulations with a model that includes key aspects of muscle dynamics, should be taken into account in the study of functional morphology and evolution of jumping animals.
